# Autonomic changes as reaction to experimental social stress in an inpatient psychosomatic cohort

**DOI:** 10.3389/fpsyt.2022.817778

**Published:** 2022-08-04

**Authors:** Carolin Thurner, Bjoern Horing, Stephan Zipfel, Andreas Stengel, Nazar Mazurak

**Affiliations:** ^1^Department of Psychosomatic Medicine and Psychotherapy, University Hospital, Tübingen, Germany; ^2^Department of Systems Neuroscience, University Medical Center Hamburg-Eppendorf, Hamburg, Germany; ^3^Clinic for Psychosomatic Medicine, Charité Center for Internal Medicine and Dermatology, Humboldt-Universität zu Berlin and Berlin Institute of Health, Charite - Universitätsmedizin Berlin, Corporate Member of Freie Universität Berlin, Berlin, Germany

**Keywords:** autonomic nervous system, cyberball, psychosomatic disorders, heart rate variability, skin conductance

## Abstract

**Objectives:**

Patients with psychosomatic disorders suffer from social isolation that might further lead to destabilization and exacerbation of bodily symptoms *via* autonomic pathways. We aimed to investigate the influence of controlled social stress (model of social ostracism) on the autonomic nerve system (ANS) in an inpatient cohort with psychosomatic disorders.

**Methods:**

We examined heart rate variability (HRV), skin conductance (SC) and skin temperature (ST) as well as ECG-derived respiration rate (EDR) and subjective reports on stress during exposure to experimental social stress (cyberball game). Data were collected from 123 participants (f:m = 88:35, 42.01 ± 13.54 years) on admission and upon discharge from the university psychosomatic clinic. All data were recorded during baseline, inclusion and exclusion phases of the cyberball game as well as during the recovery phase.

**Results:**

We found significant changes between admission and discharge with a decline in parasympathetic-related HRV parameters (SDRR −3.20 ± 1.30 ms, *p* = 0.026; RMSSD: −3.77 ± 1.28 ms, *p* = 0.007) as well as a decrease in SC (−0.04 ± 0.17 μS, *p* = 0.019) and EDR (−0.01 ± 0.01 Hz, *p* = 0.007), suggesting a drop in sympathetic tonus, with no changes in ST (*p* = 0.089) and subjective stress levels (*p* = 0.322). HRV parameters decreased during the cyberball game (SDRR *p* = 0.026; RMSSD *p* = 0.002; lnHF *p* < 0.001). In contrast, both SC (*p* < 0.001) and EDR (*p* < 0.001) increased during the game with SC being slightly lower during the exclusion phase. This can point toward a stimulation of sympathetic nervous system during game participation, which was concordant with the rise in subjective stress values (*p* < 0.001). ST showed a continuous, unspecific rise over time (*p* < 0.001).

**Conclusion:**

Our data demonstrate the decrease of ANS parameters during experimental social stress when data upon discharge were compared to those upon admission. These results are partially contradictory to previous studies that showed a rise in HRV in a psychiatric cohort over the course of (outpatient) treatment. Further research is required to help attributing these differences to effects of treatment or acute states relating to admission to or discharge from a psychosomatic department.

## Introduction

Mental disorders are regularly accompanied by changes in parameters of the autonomic nervous system (ANS), which helps explaining the higher risk for many somatic diseases in this patient group, including higher cardiovascular mortality (1–4). Some of those parameters serve both differentiating (5) and prognostic (6) purposes, especially in depressive disorders, and thus could be of interest for clinical practice to operationalize the patients' subjective feelings and to gain a more thorough understanding of therapeutic processes (7). In many studies, training to manipulate these parameters *via* biofeedback has been shown to exert a positive impact on the course of therapy using, for example, feedback of heart rate variability, skin conductance, or skin temperature (8–10).

The analysis of heart rate variability (HRV) is a well-established method of investigating ANS activity and reactivity. HRV is based on the flexibility of the heart to adapt to different psychological and physiological demands. It allows to estimate the general sympathetic and parasympathetic contributions to cardiac function (11). The general reduction of HRV, and especially of parameters related to the parasympathetic nervous system, is a frequent symptom in mental disorders (4, 12–14). This has also been observed in a wide variety of psychosomatic disorders, including depressive disorders (15, 16), anxiety disorders (17, 18), chronic pain disorders (19) and post-traumatic stress disorder (20, 21). In patients with anorexia nervosa, data are not entirely clear with most studies reporting an increased HRV (22–24), but others demonstrating no change (25, 26) or even a decrease in some parameters (27). This discrepancy has been proposed to be due to the central role underweight plays in anorexia nervosa, overshadowing effects of stress (23). Similarly, HRV at the beginning of outpatient psychotherapeutic treatment was shown to be predictive of the outcome and also increased over the course of treatment (6, 28) but did not return to normal values (29). However, in another study with a small number of participants and with group therapeutic treatment over 10 weeks, no significant change of various HRV parameters [high frequency (HF) and low frequency (LF) analysis, total power, “square root of the mean squared differences of successive RR intervals” (RMSSD), stress index] was observed (30). Although changes in HRV during outpatient treatment were investigated in various studies mentioned above, we identified only two studies that investigated HRV during inpatient psychosomatic treatment. One study found no change in HRV-parameters during inpatient treatment (31), while another detected significantly lower RMSSD at discharge with no change in other HRV parameters (32).

The literature refers skin conductance (SC), as well as the responsiveness of SC to stress, to the function of the sympathetic branch of the ANS (33). SC is lower in patients with depressive disorders than in healthy controls (34, 35) and also discriminates between patients with depression and healthy controls (5). In contrast, SC was shown to be increased in patients with post-traumatic stress disorder (21). The data on patients with other mental disorders, such as anxiety disorders, is less clear regarding SC (36, 37), with higher variance of results among studies and conditions. Some studies investigated SC in a specific therapeutic context, for example, in response to phobic stimuli during the course of exposure therapy (38), in relation to therapeutic interventions during a psychotherapeutic session (39, 40) or during relaxation therapy (41). Others used SC as a monitoring tool during hypnotherapeutic interventions (42, 43) or as predictor for clinical outcome of interventions (44). So far, studies are lacking that examined SC at rest or in exposure to social stress over the course of psychotherapy or inpatient psychosomatic treatment.

Comparing ANS parameters of patients and healthy controls, or monitoring them over the course of treatment, can give information on the general state of the ANS in patients. However, it is also possible to directly examine the effect of specific experimentally induced stressors to gain insight into specific psychological and psychosomatic connections. Most studies use classical mental stress tests such as a mental arithmetic task (31, 45) to measure the stress response. However, in most theories on mental illnesses, difficulties with and inclusion in social interactions play a much bigger role than mental stress. Relatedly, studies have shown that social inclusion is important for mental health (46). Social exclusion, however, can contribute to the development of psychiatric and psychosomatic disorders (47). The so-called Need-Threat Model suggests that repeated social exclusion threatens essential needs and—in addition to short-term negative reflexive and reflective effects—leads to resignation, helplessness and depression (48). Patients with mental illness are more sensitive to exclusion, with patients with borderline disorders in particular being highly susceptible (49), a finding consistent with clinical experience.

To simulate social inclusion and exclusion under experimental conditions, the cyberball game (50, 51) has become particularly popular in recent years. In this virtual scenario, subjects are presented with a ball-tossing game with two or three other “players,” who are controlled by the program. In general, the game simulates the conditions of being included into a social interaction, or being ostracized. It demonstrates stable results over different healthy and clinical populations and disorders and was previously widely used in patients with psychiatric disorders (49). Effects were consistent across a variety of experimental conditions (e.g., number of players, duration of exclusion, number of throws), subject characteristics (e.g., age, gender, and ethnicity) as well as repeated exposure with an average effect size of 1.5 standard deviations, as demonstrated in a 2015 meta-analysis of 120 cyberball studies (52). Notably, the effects remained even if participants knew that they were playing with a computer program instead of other, real players (53).

Studies have shown effects of participation in cyberball on parasympathetic activity, namely changes in heart rate variability. However, in most studies no significant difference was described between play phases with social inclusion and exclusion (54–57); only one study has demonstrated an increased heart rate during the exclusion phase (58). However, these studies were conducted in children with functional abdominal pain (54), healthy adolescents (57) or healthy adults (55, 56, 58). Studies investigating HRV parameters during cyberball in adult patients are missing. Similarly, participation led to a rise in overall SC (59), but no significant differences were evident between the social inclusion and exclusion phases (58), again examining healthy subjects. When examining ST, there are few and sometimes contradictory results. For example, one study showed that social exclusion in children led to an increased temperature of the tip of the nose (60), while another study in healthy subjects showed both a subjective sensation of cold and a lowered peripheral temperature at the fingertip (61).

While there are numerous studies that investigated the effect of cyberball on subjective parameters in many different conditions, only a few studies investigated the effects on ANS parameters. Moreover, the existing studies investigated either children or adolescent patient populations, or healthy adults. Patients with psychosomatic disorders already have altered baseline autonomic tension (4, 34) with concomitant increased sensitivity to social exclusion (49): A better understanding of the relationships between ANS parameters and social stress, as well as changes in ANS parameters over the course of therapy, could allow for a better understanding of psychosomatic relationships and facilitate the development of new objective methods for diagnosis and progress monitoring. Once a clearer understanding is achieved, it might be possible to develop methods to integrate ANS measurements in standard testing during the diagnostic phase and for treatment monitoring. Therefore, the aim of the present work was to test the following hypotheses: (1) Autonomic parameters such as HRV, SC and peripheral ST change over the course of inpatient psychosomatic treatment. (2) The simulation of social inclusion and exclusion with the help of the cyberball game causes an ANS response in patients with underlying psychosomatic disorders. (3) The autonomic activity and reaction to social exclusion differ between patients with different clinical diagnoses.

## Methods

### Study design and subjects

The study was approved by the Ethics Committee of the Medical Faculty of Eberhard-Karls-University (105/2019BO2). The study was conducted at the Department for Psychosomatic Medicine and Psychotherapy, University Hospital Tübingen (Universitätsklinikum Tübingen, UKT) as a prospective, uncontrolled cohort study. Participants were recruited among the patients referred to the full-time or part-time inpatient treatment in the department. Patients in part-time inpatient treatment attended the therapy program from 8 am to 4 pm Monday through Friday, did not stay overnight and spent weekends at home. All patients admitted to the Department of Psychosomatic Medicine and Psychotherapy at the UKT for inpatient treatment, who met the inclusion and exclusion criteria and gave their written consent for study participation were included in the study.

Inclusion criteria were admission to a regular inpatient or part-time inpatient treatment in the Department of Psychosomatic Medicine and Psychotherapy of the UUKT, age ≥18 years and sufficient knowledge of German language. Exclusion criteria were acute psychotic illness, brain organic disorders, current substance dependence on alcohol or illicit drugs, and patients who did not wish to participate.

For recruitment, patients were continuously screened for inclusion and exclusion criteria across all patient and diagnostic groups by the attending physician. In the case of study eligibility, written and verbal information about the study was provided. Participation in the study was voluntary; refusal or revocation of study participation as well as withdrawal during the ongoing study was possible at any time, even without giving reasons, and was not associated with any consequences for the treatment. The patients received no financial compensation. After written informed consent, patients were enrolled in the study and assigned a study number.

Data were collected at two timepoints: t0 was defined as a baseline measurement and was held within the first 3 days after the start of the treatment; t1 was performed at the end of the hospital stay and at the earliest 3 days before discharge. In the case of a setting change during treatment, e.g., a change from full-time to part-time inpatient treatment as a step-down, t1 was performed at the end of the part-time treatment. Data collection took place under standardized conditions in an office on the premises of the psychosomatic ward. Care was taken to avoid collision of the measurement with other therapies. In order to minimize circadian variances, all measurements took place between 1:30 pm and 5:30 pm. The aim of the study was to include at least *n* = 100 patients with complete study participation by measurement t1 during a maximum recruitment period of 12 months, starting in August 2019. However, due to the SARS-CoV-19 pandemic, recruitment had to be terminated prematurely in March 2020, so that only 92 patients could be completely included.

### Patients and treatment

#### Patients

Psychosomatic clinics in Germany treat patients with a variety of disorders. Apart from somatoform disorders [including somatization disorders (ICD-10 F45.0/1), somatoform autonomic dysregulation disorders (ICD-10 F45.3X) and a variety of chronic pain disorders (ICD-10 F45.4X)] the most commonly treated disorders are affective disorders [depression (ICD-10 F32.1/2, F33.1/2)], anxiety disorders (ICD-10 F40.0, F41.1/2/8) trauma-related disorders (ICD-10 F43.1) and eating disorders [anorexia nervosa (ICD-10 F50.0/1), bulimia nervosa (ICD-10 F50.2/3) and binge eating disorder (ICD-10 F50.8)]. Indication for in-patient treatment was routinely assessed by a clinical evaluator ahead of admittance during the visit of our psychosomatic ambulance. Criteria for inpatient treatment included primary diagnosis, symptom severity, loss of every-day-functionality, comorbidities and somatic consequences (e.g., electrolyte derailment, rapid weight loss or critically low weight in patients with anorexia) as well as lack of improvement in outpatient therapy, overstrain of the support system or dysfunctional surroundings. Admittance was always voluntary. An overview of somatic comorbidities can be found in Supplementary Table 1.

#### Treatment

All patients received the treatment based on our standard psychosomatic program that included individual psychotherapy, group psychotherapy, creative therapy (music therapy or art therapy), movement therapy, relaxation therapy, senior physician visit, and were in regular contact with both nurses, psychologists and doctors. Additionally, individual therapeutic elements such as social skill training, nutritional counseling, physiotherapy, occupational therapy, gardening therapy, dance therapy, biofeedback-training and family counseling with patients' partners or relatives were added if necessary. Lastly, profound somatic diagnostic using up-to-date instrumental and laboratory methods was completed.

### Experiment

#### Test procedure

The experimental procedures during t0 and t1 were similar. The patients were asked to take a comfortable position and not to talk during the experiment in order to avoid artifacts but also to avoid verbal stress release or seeking of reassurance during ostracism. Four Ag-AgCl cutanic electrodes were placed on the chest to collect ECG data. SC was collected with two electrodes placed on the distal phalanxes of the index and middle fingers of the non-dominant hand and the temperature sensor was placed on the distal phalanx of the ring finger at the same side. All data were recorded by the mean of Nexus 10 MK II device (MINDMEDIA^®^). Further details of data collection and processing are described below.

**Figure 1:**
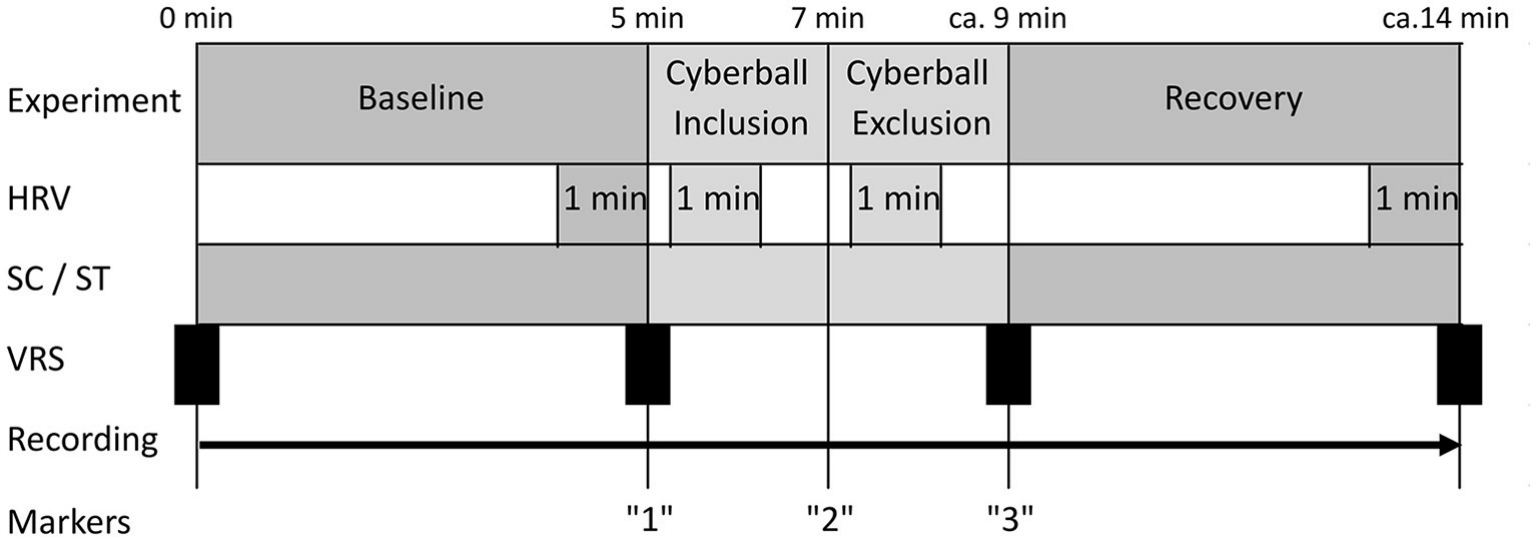
Test procedure. HRV, measured segments of heart rate variability; SC, measure segments of skin conduction; ST, measured segments of skin temperature; VRS, subjective stress level on the visual rating scale.

A baseline phase at rest of 5 min was recorded. Participants were asked to sit relaxed and avoid movements or speaking. At the end of this phase patients were instructed to start the game and recording was continued once the game began. Difficulties in the first game phase (e.g., clicking on the figure of a fellow player instead of on the picture), which hindered the progress of the game, were corrected by the experimenter, so that after 5–10 s a fluent course of the game was regularly possible. The first game phase “inclusion” lasted about 1.5–2 min (depending on patient speed and randomly assigned pauses between throws). After ~2 min the “exclusion” phase of the game started. This experimental part lasted another 1:30–2:00 min. The transition between the two phases was not disclosed to patients in any way. After the end of the game participants were asked to rest for 5 min under continued recording. Subjective stress level on a visual rating scale (VRS) with a range of 0–10 was collected prior to investigation, after baseline, after exposure to cyberball and at the end of the measurement. The duration of the entire experiment was ~20–30 min (see Figure 1).

#### Cyberball game

The cyberball game with a total of three players (patient and two computer-controlled teammates, of which the patient was told were live players on the internet) tossing a virtual ball was used to simulate social stress, while the patient was asked to visualize the game as if it was happening in real life. For the two “virtual” participants, a picture of a man and a woman was displayed with common first names assigned to them and changed regularly. Patients were told that they were playing with other real-life players over the internet and were informed of the digital simulation only after the second measurement.

The game comprised a total of 70 throws and was divided into two phases. In the first phase of the game, “inclusion,” the probability of throwing the ball to the patient was equal to the probability of passing the ball to the other player (50%). In the second phase of the game “exclusion” the patient was completely excluded from the game and the ball was only passed back and forth between the other two players (62). In both phases, there was a pause of random length between 2 and 5 s before each throw. The phases merged continuously, so that the transition could not be recognized by the patient.

### Data collected

#### Demographic data

Patient data on gender, age, clinical diagnoses and duration of treatment were collected from clinical records.

#### Autonomic parameters

All physiological data were collected by the mean of NeXus-10 MK II from MINDMEDIA^®^, medically CE certified (IIa) and FDA registered, with the associated software BioTrace +^®^, also developed by MINDMEDIA^®^. Raw ECG data were recorded at sampling rate of 256 Hz, whereas SC and PT data were recorded at a sampling rate of 32 Hz. All data were stored locally for further processing.

#### Heart rate variability

As we were mainly interested in general HRV and vagus-related HRV values during different experimental phases, four intervals each of 1 min were analyzed in the initial sample:

- Baseline: Min 04:00–05:00. This corresponds to the last minute of the baseline measurement.
- Inclusion: Min 05:15–06:15. Fifteen s after the start of the cyberball game, initial difficulties were usually over.
- Exclusion: Min 07:15–08:15. Fifteen s after the average start of the simulated social exclusion. By that time all patients had arrived in the exclusion phase, independent of playing speed and variable pause lengths.
- Recovery: Last minute of the measurement.

The use of ultra-short time sequences was investigated in several studies that compared the analysis of ultra-short time measurements with short time measurements demonstrating high correlation between measurements (63–65). Baek et al. (66) investigated the reliability of this method and concluded that ultra-short time analysis is reliable and comparable to the standard 5-min interval analysis.

HRV analysis was performed by the mean of Kubios HRV Premium 3.1 (Koopio, Finland) by experienced investigators (CT, NM). R-spikes were detected in the raw ECG signal and RR intervals were measured in milliseconds. To create the periodogram, these RR intervals (Y-axis) were plotted over the course of the measurement (X-axis). All selected intervals were screened for artifacts. The usual nature of the artifacts was incorrect R detection or signal irregularities resulting from technical or “natural” events (extrasystole). Incorrectly detected R-spikes were manually corrected. Signal irregularities were corrected using the in-build artifact correction algorhythm (67). In case the number of artifacts in selected interval was >10%, the interval was excluded from the further analysis. In total 46 (5.3%) from total 860 intervals were corrected and 77 (9.0%) were excluded from the analysis. Table 1 summarized details on calculated HRV parameters including EDR (ECG-derived respiration) (11, 68–72).

**Table 1:** Parameters calculated for analysis of heart rate variability.

**Value**	**Definition**	**Influenced by**
RR (ms)	average duration of the interval between two consecutive R-spikes	Sympathetic and parasympathetic NS
SD RR (ms)	standard deviation of the individual RR intervals from the average	Parasympathetic NS > sympathetic NS
RMSSD (ms)	square root of the mean squared differences of successive RR intervals	Parasympathetic NS
lnHF [ln (ms^2^)]	High-frequency spectral analysis with autoregressive technique of frequencies between 0.15 and 0.4 Hz, ln transformed	Parasympathetic NS
EDR (Hz)	electrocardiogram derived respiration rate	Sympathetic and parasympathetic NS

NS: nervous system.

#### Skin conductance and peripheral skin temperature

SC and peripheral ST were exported as ASCII files with a sample rate of 32 Hz. Subsequently, we calculated the mean value for each of four experimental phases: “baseline,” “inclusion,” “exclusion” and “recovery”.

### Statistical analysis

Statistical analysis was performed using IBM SPSS (IBM, Version 27.0.1.0) and MATLAB R2020b (The Math-Works Inc., Natick, MA, USA). Due to drop outs and technical issues some data were missing. Prior to statistical analysis we conducted an analysis of the missing data and performed data imputation for missing values. The latter was done by mean of the SPSS data imputation model with multiple regression sets of five. Subsequently the mean values of the imputed sets were calculated, which were used for further analysis. Data imputation was performed for all 123 patients who participated in the t0 measurement, imputing the expected values for t1 and this data set was used for the intention-to-treat (ITT) analysis. A total of 1,289 individual values (18.7%) were imputed for that purpose. The per-protocol (PP) analysis included only the 92 patients that completed the study and participated in the t1 measurement. Here, 381 (7.4%) values were imputed.

Demographic and treatment related data were calculated using descriptive statistic. In addition, the distribution of leading diagnoses in 3 diagnostic categories (affective disorders, somatoform disorders, and eating disorders) was analyzed.

Due to the hierarchical nature of the data, we used a longitudinal hierarchical linear modeling (HLM) approach for analysis of the eight key dependent variables (EDR, lnHF, RMSSD, RR, SC, SDRR, ST, VRS) (73). In the primary HLM analyses, we entered time variables (time points of treatment t0/t1, cyberball phases baseline/inclusion/exclusion/recovery) as categorical within-person predictors.

Random intercepts were included in all models. In a stepwise fashion comparing the Akaike Information Criterion (AIC) between pairs of models, we ascertained the benefit of considering random slopes for the respective predictors. Most models were considered optimal when including random slopes for both treatment and cyberball. For the intention-to-treat analysis only, predicting lnHF and EDR was considered optimal using only the treatment random effect. Detailed descriptions of HLMs and model selection are provided in Supplementary Tables 2, 3.

Secondary analyses including between-person level covariates were performed to assess the robustness of the main results, and to explore whether any of the covariates showed any effect. The covariates included person-related variables (gender, age, BMI), setting-related variables (days between t0 and t1, ward vs. day clinic setting), psychological disorders (affective, somatic symptom, eating), and current medication (tri- and tetracyclic antidepressants, SSRI/SNRIs, antipsychotics, anticonvulsants; note that only medication was considered that was used in at least 10% of the sample). Non-categorical variables were centered prior to inclusion. As with the primary analyses, inclusion of the covariates as random effects was performed given a lower AIC compared to the respective less complex model; details are provided in Supplementary Tables 4, 5. Results from secondary analyses are reported only if the respective covariate shows a significant main effect on the dependent variable. As for the robustness of the main results, we only point out if inclusion of the respective covariate changes either of the main effects of the primary predictors.

HLM analyses were performed using the MATLAB LinearMixedModel class' fitlme, compare and coefTest. The significance level was set at α = 0.05 for all tests. HLM estimates are reported as mean ± conditional predicted responses considering both fixed and random effects.

## Results

### Sample characteristics

Sample characteristics are detailed in Table 2.

**Table 2:** Sample characteristics.

**Parameter**	**Intention-to-treat total (%)** **or [mean ±SD]**	**Per-protocol total (%)** **or [mean ±SD]**
Inclusion in study	123 (100%)	
Participation in t1	92 (74.8%)	92 (100%)
Dropout	31 (25.2%)	
**Sex**		
Female	88 (71.5%)	64 (69.6%)
Male	35 (28.5%)	28 (30.4%)
**Primary setting**		
Full-time inpatient	91 (74.0%)	71 (77.2%)
Part-time inpatient	32 (26.0%)	21 (22.8%)
**Diagnostic category**		
Affective disorders	67 (54.5%)	51 (55.4%)
Somatoform disorders	47 (38.2%)	35 (38.0%)
Eating disorders	9 (7.3%)	6 (6.5%)
Age [years]	[42.01 ± 13.54]	[42.80 ± 13.49]
Time between measurements [days]	[48.45 ± 12.95]	[48.43 ± 14.96]

### Changes of autonomic data during the treatment

Over the course of treatment, there were several significant changes in autonomic data in the intention-to-treat analysis. While RR (*p* = 0.590) and lnHF (*p* = 0.079) did not differ as treatment main effects, the remaining HRV parameters dropped significantly from admission to discharge (SDRR −3.20 ± 1.30 ms, *p* = 0.026; RMSSD: −3.77 ± 1.28 ms, *p* = 0.007). Likewise, both EDR (−0.01 ± 0.01 Hz, *p* = 0.007) and SC (−0.04 ± 0.17 μS, *p* = 0.019) showed a significant drop over the course of treatment. However, ST (*p* = 0.089) and subjective stress reports (*p* = 0.322) did not change significantly (Table 3). In the per-protocol analysis, only the effect on EDR (*p* = 0.022) and SC (*p* < 0.001) remained significant (all others *p* > 0.14). Please see also Supplementary Figure 1 for distribution plots.

**Table 3:** Changes of ANS parameters and subjective stress level between assessment points on admission and discharge.

	**t0**	**t1**	***F*_(4, 976)_**	***P***
RR	810.117 ± 10.768	795.195 ± 8.107	0.703	0.590
SDRR	29.426 ± 1.220	26.229 ± 0.845	2.770	0.026*
RMSSD	26.295 ± 1.395	22.524 ± 0.886	3.556	0.007*
lnHF	5.144 ± 0.111	4.940 ± 0.088	2.099	0.079
EDR	0.273 ± 0.004	0.260 ± 0.003	3.527	0.007*
SC	2.972 ± 0.176	2.562 ± 0.145	4.301	0.002*
ST	32.609 ± 0.301	32.502 ± 0.265	2.025	0.089
VRS	2.907 ± 0.165	2.713 ± 0.122	1.170	0.322

t0, admission; t1, discharge; EDR, ECG derived respiration; lnHF, high frequency analysis; RMSSD, Square root of the mean squared differences of successive RR intervals; RR, mean interval between two consecutive RR-intervals; SC, skin conductance; SD RR, standard deviation of the difference between two consecutive RR intervals. ST, skin temperature; VRS, visual rating scale. HLM estimates are reported as mean ± SE. *significance at α = 0.05.

### Effects of social stress on autonomic parameters

**Figure 2:**
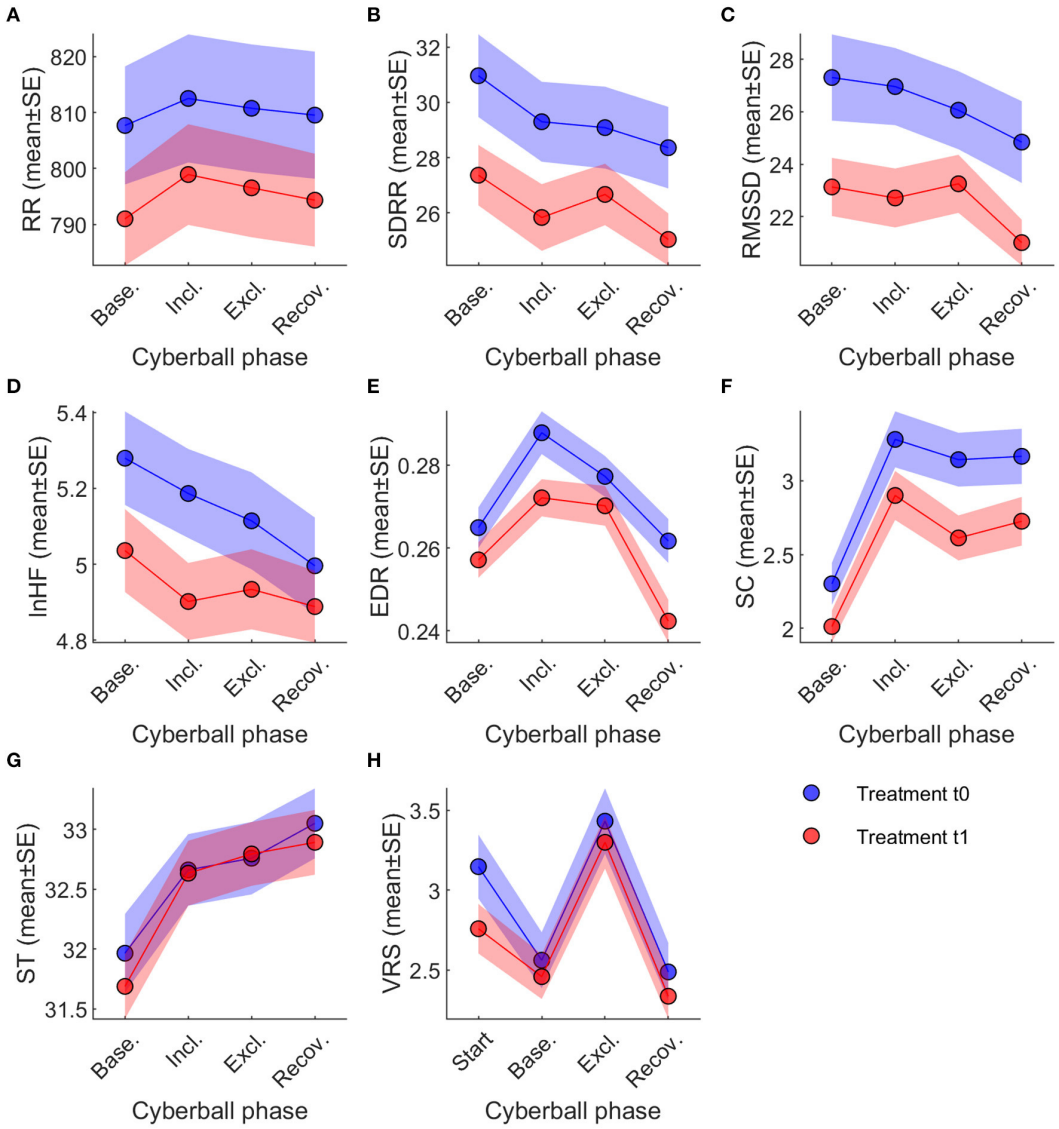
Changes of ANS Parameters under experimental social stress condition. Across-session time courses of the eight dependent variables, split by treatment t0 (blue) and t1 (red). Note that VRS was obtained at different time points than the physiological variables. Legend and abbreviations: **(A)** RR; **(B)** SDRR; **(C)** RMSSD; **(D)** lnHF; **(E)** EDR; **(F)** SC; **(G)** ST; **(H)** VRS. ANS, autonomic nervous system; EDR, ECG derived respiration; lnHF, high frequency power; RMSSD, Square root of the mean squared differences of successive RR intervals; RR, mean interval between two consecutive RR-intervals; SC, skin conductance; SD RR, standard deviation of the difference between two consecutive RR intervals; ST, skin temperature; VRS, visual rating scale (used to measure subjective stress level). Data are reported as mean ± standard error.

Intention-to-treat analysis indicated significant main effects of game participation on all parameters except for RR (*p* = 0.702, see Figure 2A; Table 4).

**Table 4:** Changes of ANS values and subjective stress during exposure to cyberball—main effects and *post-hoc*-comparisons of single phases.

**RR: Main effect of cyberball-phase:** ***F***_**(6, 976)**_ = **0.635**, ***p*** = **0.702**				
	**Baseline**	**Inclusion**	**Exclusion**	**Recovery**
Baseline		+4.833 ± 4.460 (*p* = 0.279)	+3.077 ± 4.364 (*p* = 0.481)	+1.849 ± 4.303 (*p* = 0.668)
Inclusion	+7.915 ± 4.460 (*p* = 0.076)		−1.756 ± 3.743 (*p* = 0.639)	−2.984 ± 3.935 (*p* = 0.449)
Exclusion	+5.536 ± 4.364 (*p* = 0.205)	−2.379 ± 3.743 (*p* = 0.525)		−1.228 ± 3.947 (*p* = 0.756)
Recovery	+3.329 ± 4.303 (*p* = 0.439)	−4.586 ± 3.935 (*p* = 0.244)	−2.207 ± 3.947 (*p* = 0.576)	
**SDRR: Main effect of cyberball-phase:** *F*_(6, 976)_ = **2.408**, ***p*** = 0.026*				
	**Baseline**	**Inclusion**	**Exclusion**	**Recovery**
Baseline		−1.663 ± 1.065 (*p* = 0.119)	−1.876 ± 1.067 (*p* = 0.079)	−2.601 ± 0.958 (*p* = 0.007)
Inclusion	−1.529 ± 1.065 (*p* = 0.151)		−0.213 ± 1.015 (*p* = 0.834)	−0.938 ± 1.085 (*p* = 0.388)
Exclusion	−0.695 ± 1.067 (*p* = 0.515)	+0.834 ± 1.015 (*p* = 0.412)		−0.725 ± 1.088 (*p* = 0.506)
Recovery	−2.323 ± 0.958 (*p* = 0.016)	−0.794 ± 1.085 (*p* = 0.465)	−1.627 ± 1.088 (*p* = 0.135)	
**RMSSD: Main effect of cyberball-phase:** *F*_(6, 976)_ = **3.609**, ***p*** = 0.002*				
	**Baseline**	**Inclusion**	**Exclusion**	**Recovery**
Baseline		−0.346 ± 0.901 (*p* = 0.701)	−1.249 ± 0.945 (*p* = 0.187)	−2.467 ± 0.795 (*p* = 0.002)
Inclusion	−0.421 ± 0.901 (*p* = 0.641)		−0.902 ± 0.793 (*p* = 0.255)	−2.121 ± 0.854 (*p* = 0.013)
Exclusion	+0.121 ± 0.945 (*p* = 0.898)	+0.542 ± 0.793 (*p* = 0.495)		−1.219 ± 0.892 (*p* = 0.172)
Recovery	−2.126 ± 0.795 (*p* = 0.008)	−1.705 ± 0.854 (*p* = 0.046)	−2.247 ± 0.892 (*p* = 0.012)	
**lnHF: Main effect of cyberball-phase:** *F*_(6, 976)_ = **3.944**, ***p***<0.001*				
	**Baseline**	**Inclusion**	**Exclusion**	**Recovery**
Baseline		−0.093 ± 0.069 (*p* = 0.178)	−0.165 ± 0.069 (*p* = 0.017)	−0.283 ± 0.069 (*p* <0.001)
Inclusion	−0.134 ± 0.069 (*p* = 0.052)		−0.072 ± 0.069 (*p* = 0.297)	−0.190 ± 0.069 (*p* = 0.006)
Exclusion	−0.102 ± 0.069 (*p* = 0.139)	+0.032 ± 0.069 (*p* = 0.642)		−0.118 ± 0.069 (*p* = 0.086)
Recovery	−0.148 ± 0.069 (*p* = 0.033)	−0.013 ± 0.069 (*p* = 0.847)	−0.045 ± 0.069 (*p* = 0.510)	
**EDR: Main effect of cyberball-phase:** *F*_(6, 976)_ = **16.773**, ***p***<0.001*				
	**Baseline**	**Inclusion**	**Exclusion**	**Recovery**
Baseline		+0.023 ± 0.004 (*p* <0.001)	+0.012 ± 0.004 (*p* = 0.006)	−0.003 ± 0.004 (*p* = 0.468)
Inclusion	+0.015 ± 0.004 (*p* <0.001)		−0.011 ± 0.004 (*p* = 0.018)	−0.026 ± 0.004 (*p* <0.001)
Exclusion	+0.013 ± 0.004 (*p* = 0.004)	−0.002 ± 0.004 (*p* = 0.665)		−0.016 ± 0.004 (*p* <0.001)
Recovery	−0.015 ± 0.004 (*p* <0.001)	−0.030 ± 0.004 (*p* <0.001)	−0.028 ± 0.004 (*p* <0.001)	
**SC: Main effect of cyberball-phase:** *F*_(6, 976)_ = **38.041**, ***p***<0.001*				
	**Baseline**	**Inclusion**	**Exclusion**	**Recovery**
Baseline		+0.978 ± 0.075 (*p* <0.001)	+0.840 ± 0.068 (*p* <0.001)	+0.863 ± 0.071 (*p* <0.001)
Inclusion	+0.887 ± 0.075 (*p* <0.001)		−0.137 ± 0.054 (*p* = 0.012)	−0.115 ± 0.058 (*p* = 0.049)
Exclusion	+0.600 ± 0.068 (*p* <0.001)	−0.288 ± 0.054 (*p* <0.001)		+0.023 ± 0.057 (*p* = 0.692)
Recovery	+0.713 ± 0.071 (*p* <0.001)	−0.175 ± 0.058 (*p* = 0.003)	+0.113 ± 0.057 (*p* = 0.049)	
**ST:Main effect of cyberball-phase:** *F*_(6, 976)_ = **23.055**, ***p***<0.001*				
	**Baseline**	**Inclusion**	**Exclusion**	**Recovery**
Baseline		+0.697 ± 0.106 (*p* <0.001)	+0.795 ± 0.109 (*p* <0.001)	+1.086 ± 0.120 (*p* <0.001)
Inclusion	+0.947 ± 0.106 (*p* <0.001)		+0.098 ± 0.086 (*p* = 0.253)	+0.389 ± 0.089 (*p* <0.001)
Exclusion	+1.108 ± 0.109 (*p* <0.001)	+0.161 ± 0.086 (*p* = 0.060)		+0.291 ± 0.087 (*p* <0.001)
Recovery	+1.205 ± 0.120 (*p* <0.001)	+0.259 ± 0.089 (*p* = 0.004)	+0.097 ± 0.087 (*p* = 0.266)	
**VRS: Main effect of cyberball-phase:** *F*_(6, 976)_ = **17.866**, ***p***<0.001*				
	**Start**	**Baseline**	**Stress**	**Recovery**
Start		−0.585 ± 0.126 (*p* <0.001)	+0.285 ± 0.142 (*p* = 0.046)	−0.659 ± 0.140 (*p* <0.001)
Baseline	−0.300 ± 0.126 (*p* = 0.018)		+0.870 ± 0.136 (*p* <0.001)	−0.073 ± 0.128 (*p* = 0.568)
Stress	+0.541 ± 0.142 (*p* <0.001)	+0.840 ± 0.136 (*p* <0.001)		−0.943 ± 0.136 (*p* <0.001)
Recovery	−0.422 ± 0.140 (*p* = 0.003)	−0.123 ± 0.128 (*p* = 0.339)	−0.963 ± 0.136 (*p* <0.001)	

Cyberball-phase: baseline, inclusion, exclusion, recovery; EDR, ECG derived respiration; lnHF, high frequency power; RMSSD, Square root of the mean squared differences of successive RR intervals; RR, mean interval between two consecutive RR-intervals; SDRR, standard deviation of the difference between two consecutive RR intervals; SC, skin conductance; ST, skin temperature; VRS, visual rating scale (used to measure subjective stress level). The right/top part of the individual tables presents the effects of cyberball during t0, while the bottom/left part presents the effects of cyberball during t1. In both cases, the data is presented as later-earlier phase, respectively. HLM estimates are reported as mean ± SE. ^*^significance of main effect at α = 0.05.

Pairwise comparisons of the different cyberball phases (baseline, inclusion, exclusion, recovery) indicate, however, that these main effects are driven by changes that are not uniform across parameters (Table 4; also see Figure 2). SDRR decreased between baseline and recovery during t0 (−2.60 ± 0.96, *p* = 0.007) and t1 (−2.32 ± 0.96, *p* = 0.016). RMSSD decreased at recovery from baseline (−2.47 ± 0.80, *p* = 0.002) and inclusion (−2.12 ± 0.85, *p* = 0.013) during t0; during t1, decreases at recovery were significant from baseline (−2.13 ± 0.80, *p* = 0.008), inclusion (−1.71 ± 0.85, *p* = 0.046), and exclusion phases (−2.25 ± 0.89, *p* = 0.012).

EDR showed a significant main effect (*p* < 0.001, see Figure 2E; Table 4) over cyberball exposure. All pairwise comparisons were significant except between baseline and recovery in t0 (*p* = 0.468) and between inclusion and exclusion in t1 (*p* = 0.665). Both in t0 and t1 there was a rise between baseline and inclusion (both *p* < 0.001), a drop from inclusion to exclusion only in t0 (*p* = 0.018) and a drop both in t0 and t1 from exclusion to recovery (both *p* < 0.001).

Similarly, SC analysis showed significant changes during the experimental stress condition (*p* = 0.002, see Table 4; Figure 2F). Pairwise comparisons showed a significant rise from baseline to inclusion both in t0 and t1 (both *p* < 0.001), a drop from inclusion to exclusion (t0: *p* = 0.012; t1: *p* < 0.001) and a slight rise toward recovery significant only in t1 (t0: *p* = 0.692; t1: *p* = 0.049). All other pairwise comparisons for SC were also significant (see Table 4). The differences in ST were also significant (*p* < 0.001, see Table 4; Figure 2G). Pairwise comparisons showed a continuous rise over the course of the experiment in all pairings except between inclusion and exclusion in t0 (*p* = 0.253) and in t1 between inclusion and exclusion (*p* = 0.060) and from exclusion to recovery (*p* = 0.266; see Table 4).

Subjective stress levels also changed significantly (*p* < 0.001, see Table 4; Figure 2H) at different phases of social stress provocation. *Post-hoc* analysis demonstrated significant differences for all comparisons except between baseline and recovery both in t0 (*p* = 0.568) and t1 (*p* = 0.339; Table 4). After the drop between the reports at the begin and baseline (t0: *p* < 0.001, t1: *p* = 0.018), there was a clear rise from baseline to stress (both *p* < 0.001) with a subsequent drop toward the recovery phase (both *p* < 0.001).

Per-protocol analysis indicated similar main results except RMSSD and SDRR no longer reaching significance (RMSSD: *p* = 0.086; SDRR *p* = 0.069).

Notably, the only interactions between treatment and cyberball phase were found for SC and ST. In SC, the rise from baseline to exclusion was smaller during t1 than t0 (−0.24 ± 0.08, *p* = 0.001), from baseline to recovery (−0.15 ± 0.08, *p* = 0.045), and from inclusion to recovery (−0.15 ± 0.08, *p* = 0.045). For ST, the rise from baseline to inclusion was steeper during t1 than t0 (+0.25 ± 0.12, *p* = 0.039), and from baseline to exclusion (+0.31 ± 0.12, *p* = 0.010).

### Consideration of person- and setting-related covariates

Among person-related variables in the intention-to-treat analyses, gender showed a significant main effect on ST (*p* = 0.049) without affecting the other predictors. BMI had a significant main effect on all cardiac parameters (all *p* < 0.025) as well as ST (*p* < 0.001) and VRS (*p* < 0.001). Notably, inclusion of BMI removed the significance of the cyberball main effect on SDRR (new *p* = 0.134), but conversely elevated the treatment main effects above the significance threshold for lnHF (new *p* = 0.020), ST (new *p* = 0.003), and VRS (new *p* < 0.001). Similarly, age exerted significant main effects on all dependent variables (all *p* < 0.001) except ST and VRS. Age also removed the main effects of cyberball for SDRR (new *p* = 0.08), but elevated the treatment main effect for lnHF (new *p* < 0.001). Setting itself did not have a significant effect on any of the dependent variables.

Covariation results were broadly comparable in the per-protocol analyses. Gender had no significant effect on any of the dependent variables. However, BMI showed effects on all cardiac parameters (all *p* < 0.048) as well as ST (*p* = 0.003) and VRS (*p* = 0.001); only for the prediction of VRS did BMI elevate the main effect of treatment above the threshold (new *p* < 0.001). Age exerted significant main effects on all dependent variables except ST and VRS (all *p* < 0.001), in addition to elevating main effects of treatment on SDRR (new *p* = 0.022), RMSSD (new *p* = 0.002), and VRS (new *p* < 0.001).

### Consideration of diagnosis- and medication-related covariates

When considering diagnosis, affective disorders showed significant main effects only in RR (*p* = 0.043) without changing the main effects reported above. Eating disorders hat significant effects on RR (*p* = 0.009), RMSSD (*p* < 0.001), SDRR (*p* = 0.004) and ST (*p* = 0.004). The respective analyses also elevated the treatment effect on RR (new *p* = 0.039) and ST (new *p* = 0.007) while cyberball dropped below the significance threshold in SDRR (new *p* = 0.060). All other main effects reported above remained unchanged. Somatoform disorders had no main effect on any of the dependent variables. Of the medication investigated, only medication with tri-/tetracyclic antidepressants produced a significant main effect on all HRV parameters (SDRR: *p* = 0.004, RMSSD: *p* = 0.020, lnHF: *p* = 0.380). This led to a drop below the significance threshold only for SDRR in the cyberball game (new *p* = 0.074), while other main effects as reported above remained unchanged.

Per-protocol analysis indicated similar results except a significant main effect of antipsychotic medication on EDR (*p* = 0.046) with no effect on the main results reported above. The effect on RR of both affective (*p* = 0.268) and eating disorders (*p* = 0.057) dropped below the significance threshold, while elevating the significant main effect of anticonvulsive medication on RR (*p* = 0.035) without changing the main results. With regards to SDRR, the effect of eating disorders dropped below the significance threshold (*p* = 0.165).

## Discussion

We aimed to examine the physiological state and reaction to ostracism in patients with psychosomatic disorders over the course of inpatient psychosomatic treatment. We found that HRV parameters associated with the parasympathetic nervous system (SDRR, RMSSD) as well as parameters associated with the sympathetic nervous system (SC, EDR) dropped over the course of inpatient psychosomatic treatment, while there was no significant change in RR, lnHF, ST or subjective stress levels. While HRV parameters dropped over the course of the experiment, there was no direct effect of game participation on any HRV parameters. However, both SC and EDR as well as subjective stress reports rose during game participation suggesting a stimulation of the sympathetic nervous system.

Over the course of psychosomatic treatment, HRV parameters (SDRR, RMSSD) dropped, suggesting a reduction of parasympathetic activity. This is contradictory to previous studies, that showed an increased HRV (lnHF, RMSSD) over the course of outpatient therapy (6, 28) and also an association between most psychosomatic disorders and decreased HRV (4, 12–27). Thus, a rise in HRV over the course of treatment is often expected and associated with positive effects on both self-reported and clinically observed symptoms. This suggests the need of a different explanatory model other that the effects of psychosomatic treatment. Other available data also falls short of providing an interpretation of this discrepancy. While this was not directly investigated in this study, clinical experience often reveals striking effects associated with admission to inpatient treatment, which often leads to an initial relief due to the expectation of help, removal from a conflict-prone environment and release of day-to-day duties such as work and care-taking. This could lead to de-escalation of symptoms and an increased parasympathetic activity with increased HRV at t0. On t1, respectively, patients face discharge from treatment. In this phase, clinical observations often include a rise in stress levels and anxiety associated with the return to duties and responsibilities, exposure to conflicts at home and sometimes the fear of being lost without the stabilizing environment of the psychosomatic ward. In clinical experience, this often leads to a temporary exacerbation of symptoms during this phase, which could overshadow therapeutic successes and serve as explanation of the discrepancies between this study and previous research on outpatient treatment.

SC also decreased over the course of treatment, indicating a reduced sympathicotone at t1. This is generally associated with a reduction of emotional stress. However, reduced SC has also been associated with depressive disorders (34, 35), which was the main diagnosis for many patients and comorbid diagnosis for several others, although the association with other psychosomatic illnesses is not as clear (21, 36, 37). As longitudinal data for SC in general is scarce, interpretation of reduced SC is difficult to interpret in the absence of a control group. A longitudinal study examining the effect of regular meditation practice on physiological parameters outside of meditation observed reduced SC in meditation practitioners without significant changes in the control group (74), which may suggest the effect being attributable to changes in the patients' state as opposed to a simple effect of repetition. It is possible that the changes we observed are the effect of psychosomatic treatment, including relaxation techniques, giving rise to increased relaxation. This idea would be supported by other studies showing a decrease of sympathetic activity with the use of other relaxation techniques such as yoga (75), breathing exercises (76) and autogenic training (77). However, they could also hint at an aggravation of depressive symptoms for reasons discussed above. Interestingly, subjective stress perception did not change. Taken together, these results could most likely be interpreted as effects of acute states in relation to admission and discharge leading to an aggravation of (depressive) symptoms, while an effect of therapy with the regular practice of relaxation techniques could also play a role in the drop of SC. Additionally, respiratory rate was lower at t1, which may suggest reduced sympathetic nervous activity (72).

In summary we can conclude that autonomic parameters do change significantly over the course of inpatient psychosomatic treatment with a general drop both in parasympathetic and sympathetic activity. Interpretation of these changes in relation to psychosomatic disorders or inpatient treatment, however, is challenging and more research is needed—desirably with the addition of a control group and/or repeated measurements over the course of treatment to control for effects related to admission to and dismissal from therapy.

With regard to our second hypothesis, we could clearly see the rise in subjective stress levels after exposure to cyberball. This has been observed many times before (52), confirming that the task fulfilled its purpose. We observed several significant changes in autonomic data over the course of cyberball game. HRV (SD RR, RMSSD, lnHF) dropped significantly both in t0 and t1, signaling a decrease in parasympathetic activity which relates to an increase in stress. This held true for all HRV parameters when baseline and recovery were compared. Overall, there was no specific change of HRV during game participation (inclusion and exclusion) but rather a relatively continuous decline of HRV parameters over the course of the experiment, so it is difficult to attribute this to a specific effect of exposure to cyberball, instead of the experimental setting. However, it could be argued that exposure to the cyberball game leads to a steady decline in HRV, which continued on into the recovery phase suggesting a continuous rise in stress levels throughout the experiment. A previous study on HRV during the cyberball game in adolescent patients with functional abdominal pain found contradicting results in patients with increased HRV during exposure to the cyberball game, but not in controls (54). Another study on healthy subjects detected no difference between social inclusion and exclusion (55), which is in line with our current results. It is possible that the reaction observed in youths with functional abdominal pain is specific to that particular diagnosis and age group and not visible in adults with psychosomatic disorders, especially since all HRV parameters are dependent on age (78, 79). Since youths have higher baseline HRV, reaction to cyberball might differ as well.

We could, however, detect a distinct effect of game participation on EDR, which was higher during game participation, with a drop toward the exclusion phase only significant in t0, suggesting a stress reaction induced by increased sympathetic activity (72) during task performance. Additionally, SC rose during game participation, adding evidence to an increased sympathetic tone, although in this case there was no drop during recovery and even a slight rise in t1, suggesting a slower recovery after exposure to the cyberball game, which is in line with previous findings obtained in healthy subjects (58, 59). Moreover, there was a drop in SC during the exclusion phase of the cyberball game which was more dominant in t1 than in t0. This could be speculated to be the result of relief, as patients no longer had to show active participation in throwing the ball, but were allowed to observe the other players passively. This is especially interesting, as a previous study found no difference between game phases on healthy subjects (58), which could point toward a different reaction to exclusion from the cyberball game with psychosomatic patients preferring a more passive role. Subjective reports on stress after exposure to the cyberball game add to the picture of general stress reaction, as they were higher after exposure than at baseline (however, the study design does not allow for discrimination in this between inclusion and exclusion). Previous studies found a drop in peripheral skin temperature on exposure to stress (80, 81) which contradicts our current findings.

Overall, with regard to our second hypothesis, the effect of participation in the cyberball game on parasympathetic activity as measured by HRV is unspecific with no visible effects on game participation and a continuous drop in HRV over the course of the experiment, which is mostly significant in the comparison between baseline and recovery. Sympathetic reaction, however, as measured by EDR and SC, was clearly visible during game participation, with a decrease both in SC and partially in EDR during the exclusion phase but different reactions during recovery with a clear drop in EDR and either no difference or a slight rise in SC. Subjective stress levels show a clear rise after game participation with a drop to baseline levels after recovery, which seems mirrored in sympathetic reaction, especially breathing rate, and is in line with previously shown effects on subjective stress reaction (52). For more comprehensive interpretation of the described effects, further research, especially the addition of a control group but also the randomization of game phases to control for order bias could be helpful.

There was only one specific effect of gender with men showing significantly higher ST than women. This phenomenon is well-known (82) and not likely to be related to a difference in reaction to the experimental setting or treatment. We also observed an effect of BMI and age on most of investigated variables that has already been documented elsewhere (83, 84). Interestingly, adding these covariates to our models impacted the significance of treatment main effect for lnHF, again pointing toward the close connection between parasympathetic regulation and somatic variables (85, 86). Moreover, covariate analyses indicated an effect of eating disorders on most of the HRV parameters. This issue was addressed by many researchers including ourselves; it is suggested to be related to activation of the parasympathetic branch due to restrictive eating and low body weight (22, 87). The effect of tricyclic antidepressants on HRV parameters has also been described in previous research (88), while inclusion of this covariate does not alter our current main results. As there was no difference between patients in part-time and full-time inpatient treatment, the analysis of both groups together is legitimate.

In conclusion we observed that both parameters of sympathetic and parasympathetic activity decrease significantly over the course of inpatient psychosomatic treatment in a naturalistic psychosomatic patient population, which stands in contrast to previous findings reporting over the course of outpatient psychotherapy. Further research is needed to help in interpretation of these results and to attribute specific effects to treatment itself, progress in the psychotherapeutic process and specific effects related to admission to and discharge from the inpatient treatment. Additionally, we detected a heightened reaction of mostly the sympathetic nervous system and subjective stress levels to exposure with cyberball, with evidence pointing toward lowered sympathetic activity during the exclusion phase.

### Strengths and limitations

Up to this point, the current study is the only one to our knowledge investigating the relation between social stress and ANS in inpatient cohort of patients with psychosomatic disorders. We used a highly standardized procedure in a relatively large naturalistic clinical sample modeling a social ostracism—a condition contributing to the course of psychosomatic disorders—that was barely addressed in this patient cohort in the past. The biggest limitation is the lack of a control group, which makes the comparison to healthy subjects difficult. Additionally, as mentioned above, the measurement of ANS parameters only at admittance and discharge makes it difficult to differentiate effects of treatment and changes related to the specific situation patients were in at the time, or simply repetition of the task. The other limitation of the study is the lack of age- and gender-matching between different diagnostic groups. Given that the effects of the task stayed the same in almost all instances, we do not believe this to be a serious issue. As we did not evaluate whether participants believed the cover story of participation in the cyberball task, it is possible that a lack of belief in that cover story changed the effectiveness of the task. However, previous studies have found comparable strong effects of the task even if participants knew that they were playing with a computer (53). We also discussed the possible effects of using ultra-short-time measurement for HRV with relatively short periods of 1 min in each segment. For reasons discussed above and with evidence clearly supporting the reliability of results of this segment length, we also believe this issue to be relatively minor. As the therapy concept at a psychosomatic ward is relatively specific to the German health care system, the generalizability to treatment systems in other countries might be limited. We chose a longitudinal, experimental design with a low threshold for participation in order not to dissuade more severely impaired patients from participation. Still, as sicker patients as well as patients with eating disorders often had more difficulties adjusting to the inpatient setting, these patient groups more often declined study participation. This fact led to a bias in subject selection. Due to the patients' familiarity with the experimental procedure, a habituation effect to the intervention with the cyberball game at t1 could be assumed. However, previous studies have shown that the effects of the game remain stable over time and when the experiment is repeated (89) so we did not expect habituation effects to play an important role. Additionally, the differences between inclusion and exclusion phases could be the result of order bias, as the exclusion phase was second for all patients. Further research is needed with the addition of a control group and multiple measurements over the course of treatment or with the additional measurement of changes in symptom severity or treatment satisfaction measured by psychometric instruments.

## Data availability statement

The raw data supporting the conclusions of this article will be made available by the authors, without undue reservation.

## Ethics statement

The studies involving human participants were reviewed and approved by Ethics Committee of the Medical Faculty of Eberhard-Karls-University of Tübingen. The patients/participants provided their written informed consent to participate in this study.

## Author contributions

CT was responsible for data collection, preparation of data for analysis, preliminary data analysis, data interpretation, and mainly drafted the paper. NM was responsible for conception, design, preparation of the study, drafting the paper, assisted and advised in data analysis, and interpretation. BH performed the main data analysis and presentation, was involved in the data interpretation, and drafted the paper. AS was responsible for conception and design of the study as well as data interpretation. SZ was responsible for conception and design of the study. All authors revised the manuscript and approved the final version.

## Conflict of interest

The authors declare that the research was conducted in the absence of any commercial or financial relationships that could be construed as a potential conflict of interest.

The reviewer SS declared a shared affiliation with the author AS to the handling editor at the time of review.

## Publisher's note

All claims expressed in this article are solely those of the authors and do not necessarily represent those of their affiliated organizations, or those of the publisher, the editors and the reviewers. Any product that may be evaluated in this article, or claim that may be made by its manufacturer, is not guaranteed or endorsed by the publisher.

## Supplementary material

Supplementary Table 1 Somatic comorbidities and medication.

Supplementary Table 2 Model Parameters of Intention-to-Treat Main Analysis. Summary of Intention-to-Treat Hierarchical Linear Model Comparisons Used to Determine Inclusion of Random Slopes for Treatment or Cyberball, in Addition to Fixed Effects and Random Intercept. Two Comparisons Were Performed per Dependent Variable, Comparing Model Number 1–2, and Model Number 2–3. Positive Delta AIC Indicates Superiority of the Higher (More Complex) Model, Negative the Converse. The Model With the Lower AIC Was Selected; Note That Models With Very low Delta AIC (Below 2) Are Considered Roughly Equivalent. EDR, ECG Derived Respiration; LnHF, High Frequency Power; RMSSD, Square Root of the Mean Squared Differences of Successive RR Intervals; RR, Mean Interval Between two Consecutive RR-Intervals; SC, Skin Conductance; SDRR, Standard Deviation of the Difference Between two Consecutive RR Intervals; ST, Skin Temperature; VRS, Visual Rating Scale (Used to Measure Subjective Stress Level).

Supplementary Table 3 Model Parameters of per-Protocol Main Analyses. Summary of per-Protocol Hierarchical Linear Model Comparisons Used to Determine Inclusion of Random Slopes for Treatment or Cyberball, in Addition to Fixed Effects and Random Intercept. Two Comparisons Were Performed per Dependent Variable, Comparing Model Number 1–2, and Model Number 2–3. Positive Delta AIC Indicates Superiority of the Higher (More Complex) Model, Negative the Converse. The Model With the Lower AIC Was Selected; Note That Models With Very low Delta AIC (Below 2) Are Considered Roughly Equivalent. EDR, ECG Derived Respiration; LnHF, High Frequency Power; RMSSD, Square Root of the Mean Squared Differences of Successive RR Intervals; RR, Mean Interval Between two Consecutive RR-Intervals; SC, Skin Conductance; SDRR, Standard Deviation of the Difference Between two Consecutive RR Intervals, ST: Skin Temperature; VRS, Visual Rating Scale (Used to Measure Subjective Stress Level).

Supplementary Table 4 Model Parameters of Intention-to-Treat Covariate Analyses. Summary of Intention-to-Treat Hierarchical Linear Model Comparisons Used to Determine Inclusion of Random Slopes for Covariates, in Addition to Fixed Effects, Random Intercept, and Random Slopes as in Supplementary Table 2. Comparisons Were Performed Between not Considering a Random Slope of the Covariate in Addition to the Base Models, and Considering a Random Slope. Positive Delta AIC Indicates Superiority of the Higher (More Complex) Model, Negative the Converse. The Model With the Lower AIC Was Selected; Note That Models With Very low Delta AIC (Below 2) Are Considered Roughly Equivalent. EDR, ECG Derived Respiration; LnHF, High Frequency Power; RMSSD, Square Root of the Mean Squared Differences of Successive RR Intervals; RR, Mean Interval Between two Consecutive RR-Intervals; SC, Skin Conductance; SDRR, Standard Deviation of the Difference Between two Consecutive RR Intervals; ST, Skin Temperature; VRS, Visual Rating Scale (Used to Measure Subjective Stress Level).

Supplementary Table 5 Model Parameters of per-Protocol Covariate Analyses. Summary of per-Protocol Hierarchical Linear Model Comparisons Used to Determine Inclusion of Random Slopes for Covariates, in Addition to Fixed Effects, Random Intercept, and Random Slopes as per Supplementary Table 4. Comparisons Were Performed Between not Considering a Random Slope of the Covariate in Addition to the Base Models, and Considering a Random Slope. Positive Delta AIC Indicates Superiority of the Higher (More Complex) Model, Negative the Converse. The Model With the Lower AIC Was Selected; Note That Models With Very low Delta AIC (Below 2) Are Considered Roughly Equivalent. EDR, ECG Derived Respiration; LnHF, High Frequency Power; RMSSD, Square Root of the Mean Squared Differences of Successive RR Intervals; RR, Mean Interval Between two Consecutive RR-Intervals; SC, Skin Conductance; SDRR, Standard Deviation of the Difference Between two Consecutive RR Intervals; ST, Skin Temperature; VRS, Visual Rating Scale (Used to Measure Subjective Stress Level).

Supplementary Figure 1 Distribution Plots. EDR, ECG-Derived Respiration; HF, High Frequency Power; RMSSD, Square Root of the Mean Squared Differences of Successive RR Intervals; RR, Mean Interval Between two Consecutive RR-Intervals; SC, Skin Conductance; SDRR, Standard Deviation of the Difference Between two Consecutive RR Intervals. ST, Skin Temperature; VRS, Visual Rating Scale (Used to Measure Subjective Stress Level).
