# Supplementary material for: Autonomic changes as reaction to experimental social stress in an inpatient psychosomatic cohort
Source: Front Psychiatry. 2022 Aug 4;13:817778. doi: 10.3389/fpsyt.2022.817778 (PMC9385984; doi:10.3389/fpsyt.2022.817778)

Supplemental figure 1 – Distribution Plots

a) Distribution of RR

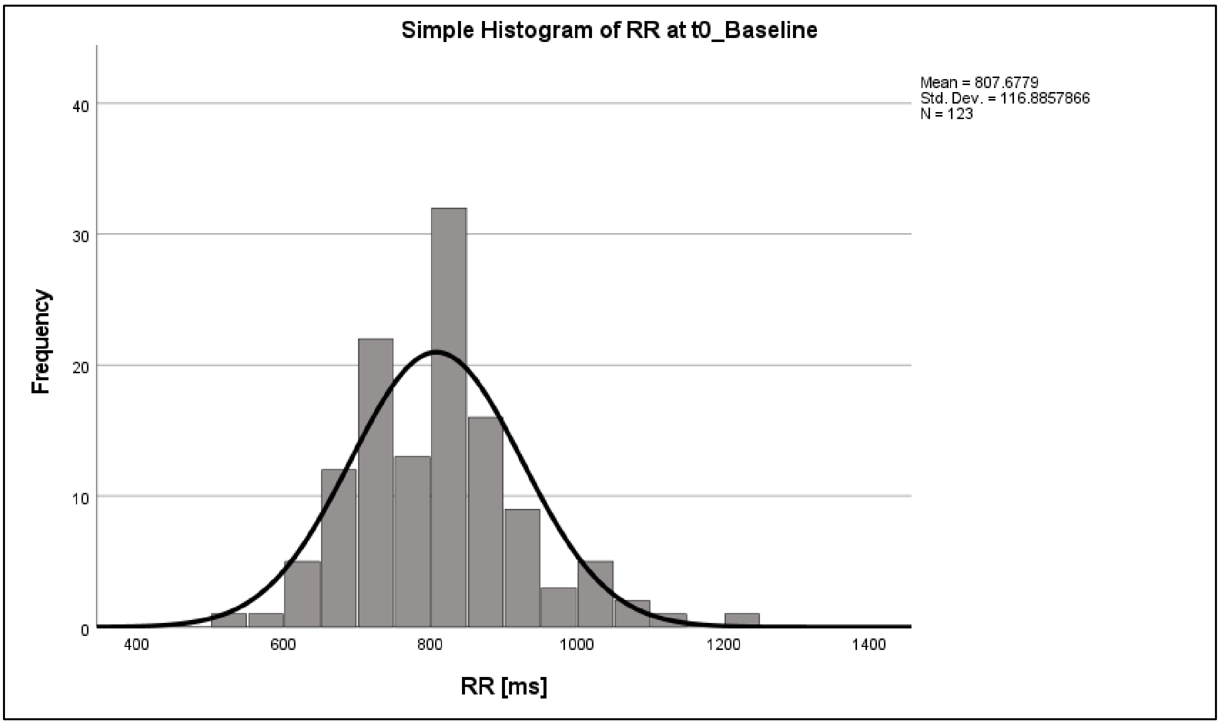

b) Distribution of SDRR

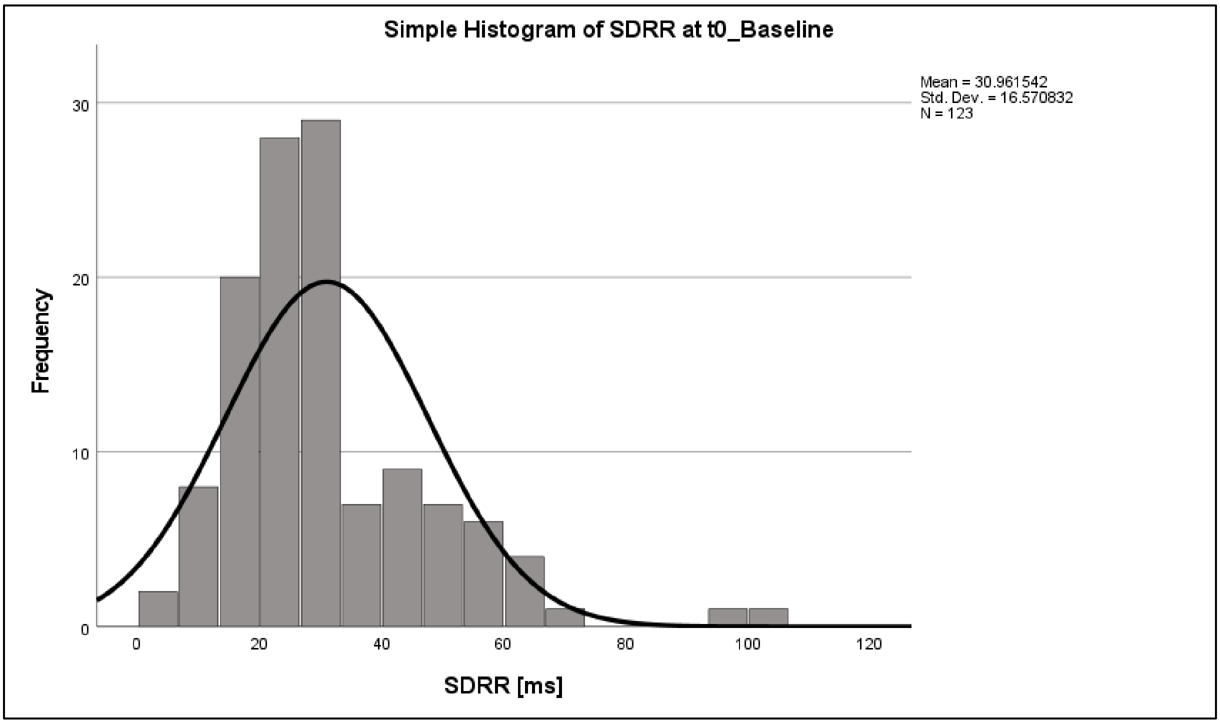

c) Distribution of RMSSD

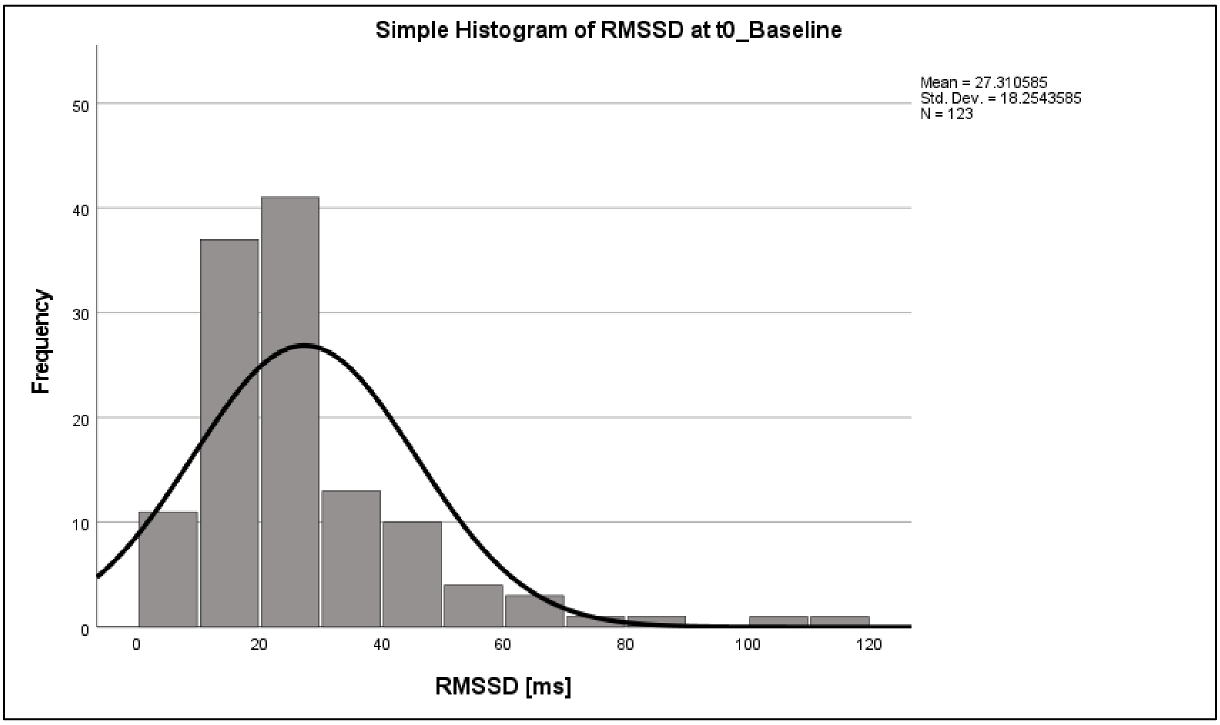

d) Distribution of HF

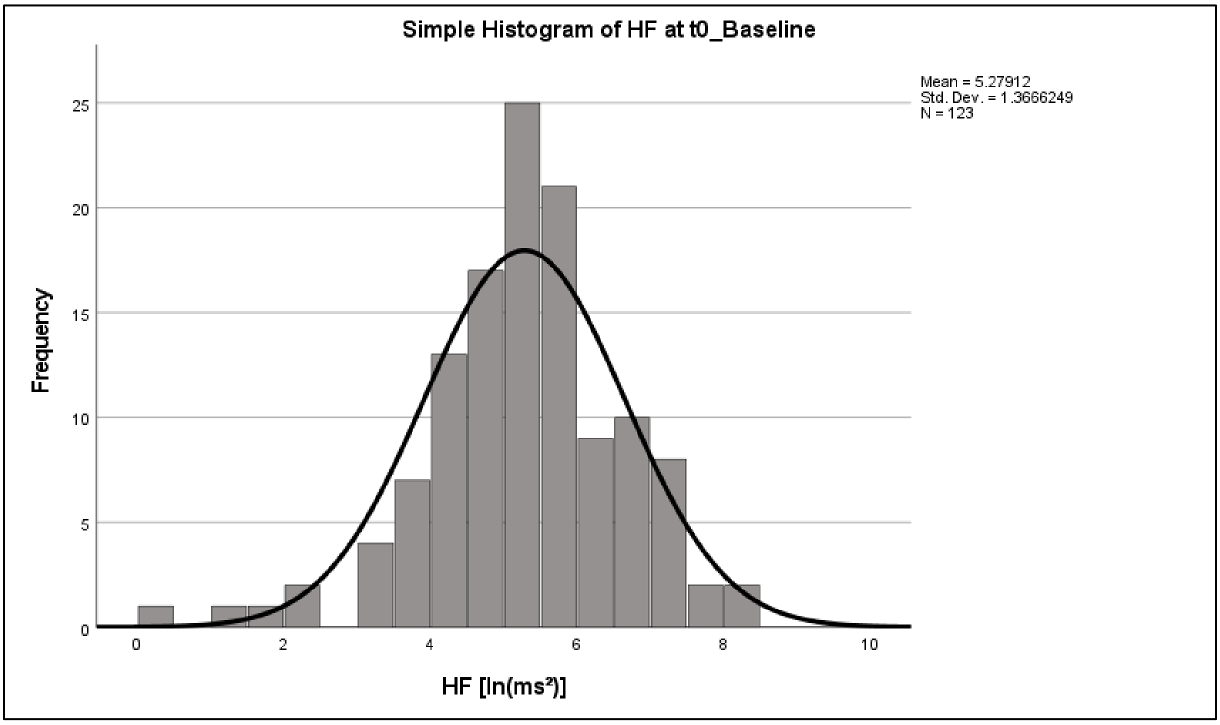

e) Distribution of EDR

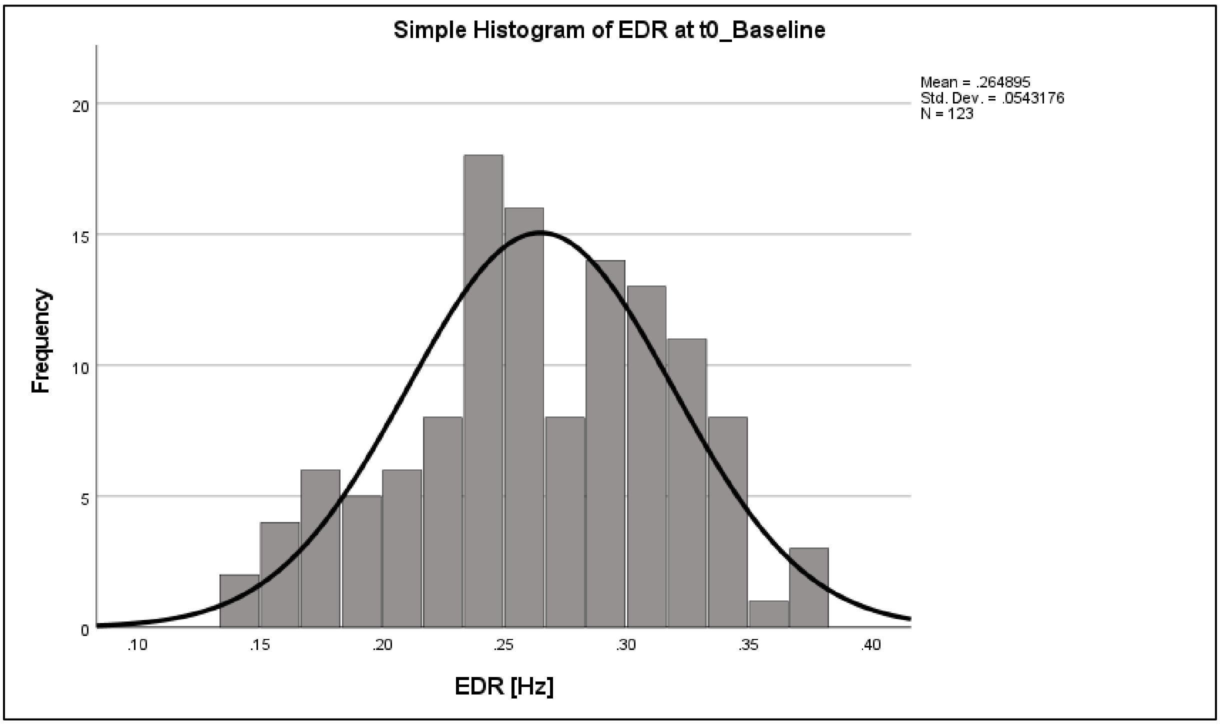

f) Distribution of Skin Conductance

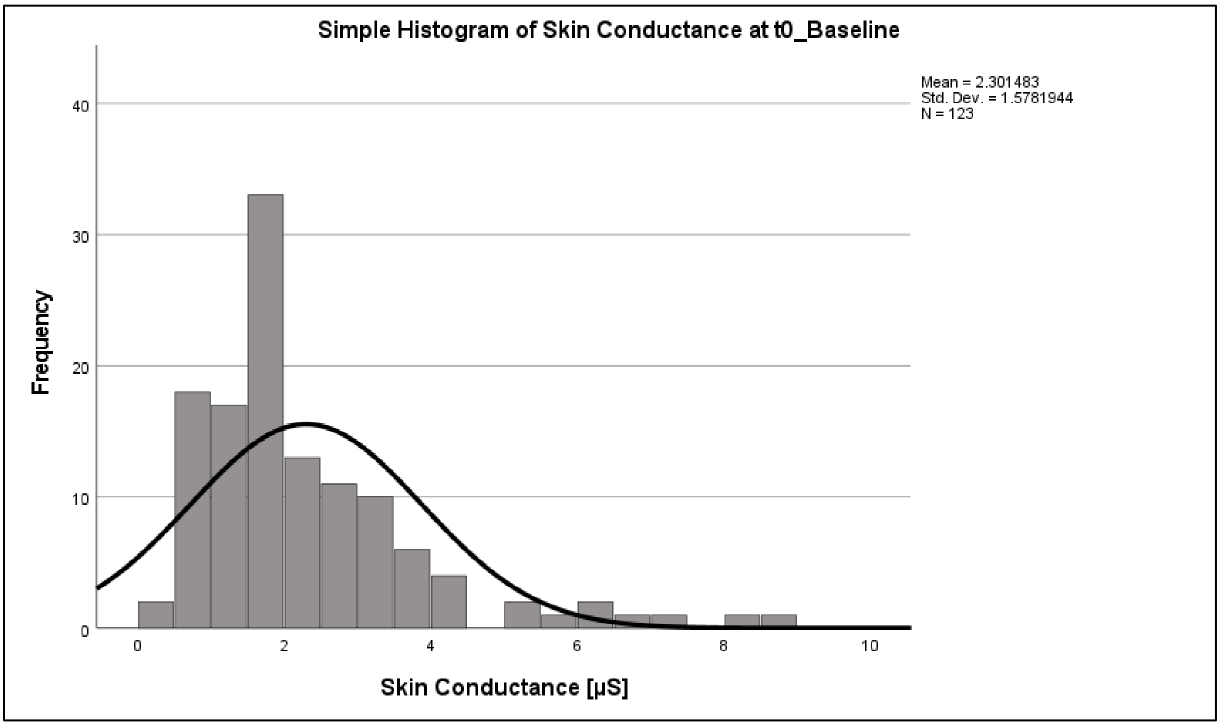

g) Distribution of Skin Temperature

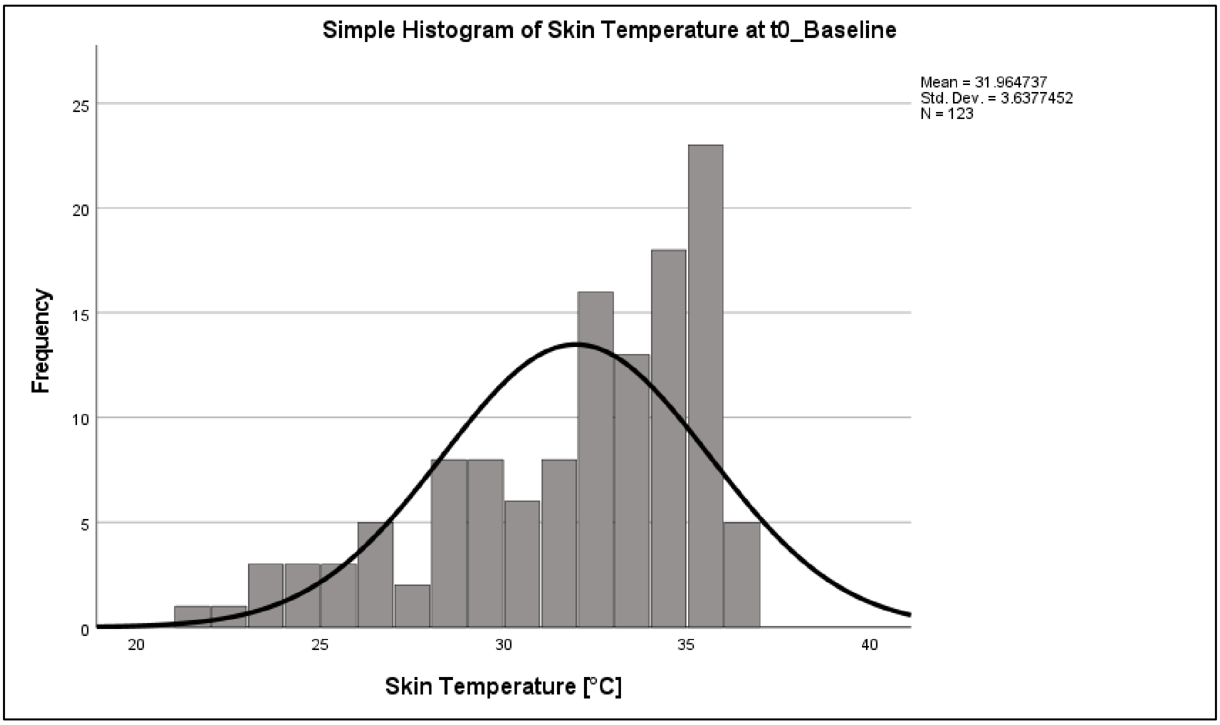

h) Distribution of Subjective Stress on VRS

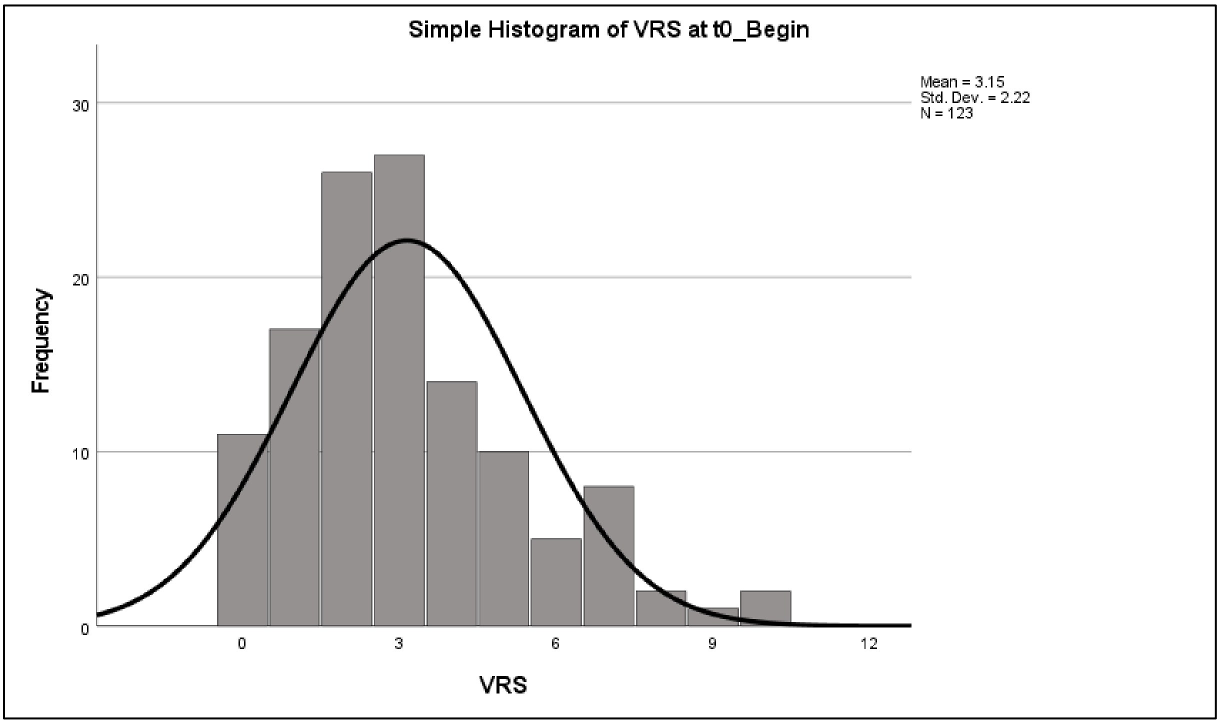

Supplement: Supplementary Figure 1 — Distribution Plots. EDR, ECG-Derived Respiration; HF, High Frequency Power; RMSSD, Square Root of the Mean Squared Differences of Successive RR Intervals; RR, Mean Interval Between two Consecutive RR-Intervals; SC, Skin Conductance; SDRR, Standard Deviation of the Difference Between two Consecutive RR Intervals. ST, Skin Temperature; VRS, Visual Rating Scale (Used to Measure Subjective Stress Level). [file Data_Sheet_1.PDF]
